# Dyslipidemia and Fatty Liver Disease in Overweight and Obese Children

**DOI:** 10.1155/2018/8626818

**Published:** 2018-06-12

**Authors:** Asma Deeb, Salima Attia, Samia Mahmoud, Ghada Elhaj, Abubaker Elfatih

**Affiliations:** ^1^Pediatric Endocrinology Department, Mafraq Hospital, P.O. Box 2951, Abu Dhabi, UAE; ^2^Pediatric Clinic, Police Health Services, Abu Dhabi, UAE; ^3^Primary Health Care Center, BaniYas, Abu Dhabi, UAE; ^4^Biochemistry Department, Shaikh Khalifa Medical City, Abu Dhabi, UAE

## Abstract

**Introduction:**

Obesity is a worldwide concern. It is associated with morbidity such as dyslipidemia and liver disease. Childhood obesity has dramatically increased, particularly in the Gulf region. We aim to assess the prevalence of dyslipidemia and fatty liver disease (FLD) in overweight and obese children and analyze the association between different anthropometric measures with dyslipidemia and fatty liver disease.

**Methods:**

A descriptive, cross-sectional study conducted on children referred with obesity. BMI percentiles were plotted and standardized waist circumference (WC) was generated. Family history of metabolic syndrome was recorded. Fasting lipid, liver transaminases, and ultrasound scans (US) for those with elevated enzymes were performed. Descriptive statistics were used for quantitative parameters.

**Results:**

216 participants were recruited. Mean ± SD age was 10.58 ± 2.996 years. 55.3% had dyslipidemia; 11.7% had high cholesterol, 28.6% high triglyceride, 32.7% high LDL, and 18.0% low HDL. 51 (84%) had either elevated transaminases. All had liver US, and 43 had FLD. WC was strongly associated with dyslipidemia and FLD (*P*=0.04  and  0.003).

**Conclusion:**

Dyslipidemia is common in overweight, obese children. FLD is prevalent in those with elevated liver transaminases. WC is an easy tool that can be utilized to screen for dyslipidemia and FLD in overweight and obese children.

## 1. Introduction

Obesity is an increasing health concern worldwide. It is strongly linked to morbidity and mortality through its associated health risks. Childhood obesity has dramatically increased to reach an epidemic proportion particularly in the Gulf area [1]. Sedentary life style led to emergence of obesity and the metabolic syndrome at a much younger age compared to the past which has been particularly pronounced in the Gulf area [2]. In a study by Freedman et al., 70% of obese or overweight children have at least one clinical cardiovascular risk factor; dyslipidemia, high blood pressure, or insulin resistance [3]. With the increasing prevalence of obesity, various forms of comorbidities are seen in children including dyslipidemia [4] and nonalcoholic fatty liver disease (NAFLD) [5]. Liver disease and dyslipidemia are major comorbidities for obesity at a young age [6, 7].

NAFLD is one of the most common causes of chronic liver disease associated with obesity worldwide. In 2016, NAFLD accounted for 20%–30% prevalence in Western countries and 5%–18% in Asia. The overall incidence of NAFLD in children has reached approximately 10% of which, 17% are teenagers and 40%–70% among obese children [8]. Childhood NAFLD may occur in young children. In those, the disease is usually asymptomatic with the diagnosis frequently made upon abdominal imaging [9].

Various surrogate markers have been used for dyslipidemia and fatty liver disease. Alanine aminotransferase (ALT) enzyme is known to reflect NAFLD and is used as the basis of performing liver biopsy when NAFLD is suspected [10].

Abnormalities in lipid metabolism in youth have shown an increased incidence around the world and are strongly associated with cardiovascular disease. High level of cholesterol has been reported among children and adolescents in the United States [11]. Similarly, 32% of children aged 11.4 ± 0.97 years old studied were found to have dyslipidemia in Chile [12].

Body mass index (BMI) and waist circumference (WC) are the most commonly used anthropometric measures to predict metabolic disorders related to childhood obesity [13]. As body fat distribution plays an important role in obesity-related comorbidities, WC is a commonly used parameter. In European boys and girls, Savva et al. established that WC has a better prediction for BP, HDL, and LDL than BMI [14].

While international data on dyslipidemia and nonalcoholic fatty liver disease (NAFLD) in obese children are abundant, there is paucity of studies in this field in the Arab countries and the Middle Eastern region where childhood obesity is common.

The aim of the study is to assess the prevalence of dyslipidemia and fatty liver disease in overweight and obese children. We also aim to analyze the association between various anthropometric measures and dyslipidemia and NAFLD.

## 2. Methods

This is a descriptive, observational, cross-sectional study conducted on children and adolescents referred for obesity management. The study is undertaken at the Paediatric Endocrinology Clinic, Mafraq Hospital, Abu Dhabi, United Arab Emirates. Overweight and obese children and adolescents referred for obesity are invited to participate in the study. Fulfilling the following criteria was ensured prior to enrolment of participants.

### 2.1. Inclusion and Exclusion Criteria

Patients referred to the Paediatric Endocrinology Clinic for obesity, having BMI above the 85th percentile and agreed to participate in the study were considered eligible. Children already diagnosed with diabetes or other hormonal disorders or those who are on long-term use of corticosteroids or antiepilepsy medication are considered unsuitable for the study.

### 2.2. Study Procedure

Patients who fulfilled the above criteria were invited to enroll in the study. Those who agreed to participate signed an assent form, and parents/guardians signed a consent form. The study was explained to participants, and they were given a study information sheet. The study is approved by the Mafraq Hospital Research and Ethics Committee.

Demographic variables including gender, age, height, weight, BMI, and waist circumference were taken. BMI was calculated as weight in kg/height^2^ in meters. SDS for weight, height, and BMI were calculated. BMI percentile above the 85th and 95th were used to define overweight and obese, respectively [15]. Waist circumference was taken at the midway point between the anterior superior iliac spine and the lower rib cage and is measured by 2 study members (AD and SA). Standardized scores for waist circumference were generated based on Jackson et al. for WC percentile in Kuwaiti children and adolescents [16]. Blood pressure was taken using appropriate cuff size for age and recorded as percentile for age and sex. Detailed family history of obesity, dyslipidemia, heart disease, and diabetes in first-degree relative was taken.

Fasting blood sample was obtained from participants for lipid profile (total cholesterol, LDL, HDL, and triglyceride) and liver transaminases: alanine aminotransferase (ALT) and aspartate aminotransferase (AST). Dyslipidemia was diagnosed if any of the lipid profile parameters are found to be abnormal as follows: total cholesterol (TC) ≥ 5.2 mmol, triglyceride (TG) > 1.5 mmol, low density lipoprotein (LDL)> 2.84 mmol, or high density lipoprotein (HDL) < 0.9 mmol. Those who had elevated liver enzyme had a liver ultrasound scan (US).

### 2.3. Laboratory Methods

Serum ALT and AST measurement was performed on the Roche/Hitachi Cobas c702 analyzer (Roche Diagnostics GmbH, Sandhofer Strasse 116, D-68305 Mannheim) to an IFCC standardized assay that was optimized for performance and stability without pyridoxal phosphate activation. The assay has an analytical Measuring range of 5–700 IU/L, a lower detection limit (analytical sensitivity) of 5 IU/L, a within run CV (%) of 3.1% at an ALT and AST of 21.5 U/L, and a between run CV (%) of 0.6% at an ALT of 546 U/L.

Total serum total cholesterol (TC) measurement was performed on the Roche/Hitachi Cobas c702 analyzer (Roche Diagnostics GmbH, Sandhofer Strasse 116, D-68305 Mannheim) with an enzymatic, colorimetric assay. The assay has an analytical measurement range of TC 0.1–20.7 mmol/L (3.86800 mg/dL), a lower detection limit of 0.1 mmol/L (3.86 mg/dL), and a within run CV (%) of 0.6% at a TC of 2.72 mmol/L and a between run CV of 0.6% at TC of 17.9 mmol/L.

Serum lipoprotein density-cholesterol measurement was performed with the Plus- 3rd generation lipoprotein-cholesterol assay on the Roche/Hitachi Cobas c702 analyzer (Roche Diagnostics GmbH, Sandhofer Strasse 116, D-68305 Mannheim). The assay was a direct homogeneous enzymatic colorimetric enzymatic assay.

Serum Triglycerides were measured by an enzymatic colorimetric assay based on the Wahlefeld method using a lipoprotein lipase performed on the Roche/Hitachi Cobas c702 analyzer (Roche Diagnostics GmbH, Sandhofer Strasse 116, D-68305 Mannheim). The assay has an analytical measurement range of 0.1–10.0 mmol/L (8.85–885 mg/dL) and a lower limit of measurement of 0.1 mmol/L, a within run CV of 0.9 (%) at a TRIGL of 1.32 mmol/L, and a between run CV (%) of 0.60% at a TRIGL of 9.21 mmol/L.

### 2.4. Statistical Methods

Data were examined graphically and numerically. Descriptive statistics were used for quantitative parameters such as mean, standard deviation, minimum, maximum, and median. Frequency and percentages are used for qualitative parameters. Bivariate logistic regressions were used to examine the relationship between variables.

Statistical difference is considered significant if *P* value is found to be ≤0.5. Statistical analysis was performed using IBM SPSS software version 22.

## 3. Results

216 patients were enrolled in the study (121, 56% males). The overall age of the sample ranged between 4 and 19 years. Mean ± SD age was 10.58 ± 2.97 years. Standardized mean ±SD for height was 1.12 ± 0.44, while that for weight was 2.30 ± 0.70. 201 (93.0%) of the children were obese with a BMI ≥ 95th percentile while 15 (7.0%) were overweight.

On physical examination, a total of 138 (63.9%) of patients had acanthosis nigricans. The standardized mean ± SD for waist circumference was 2.31 ± 1.058 cm. 173 patients (89.6%) had a WC ≥ 90th percentile. The standardized mean ± SD for systolic blood pressure was 1.10 ± 1.014 mmHg while that for diastolic blood pressure was 0.55 ± 0.812 mmHg (Table 1).

### 3.1. Dyslipidemia

**Figure 1:**
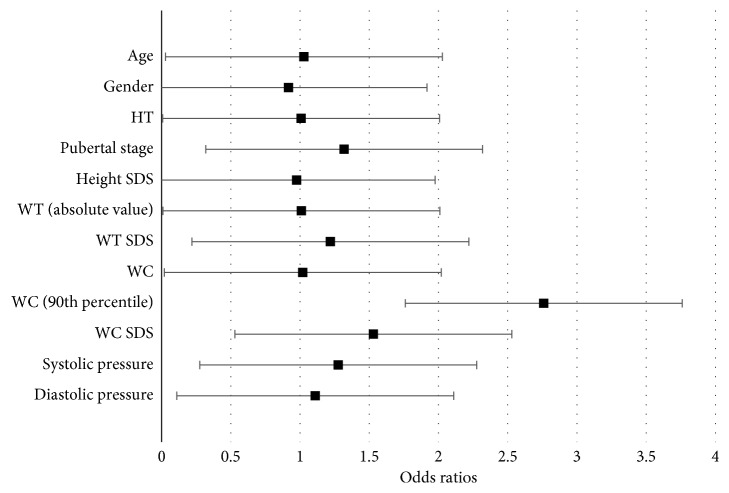
Bivariate logistic regressions between predictor variables and dyslipidemia.

Among the total sample, 55.3% of patients had dyslipidemia. 11.7% patients had high cholesterol, 28.6% had high triglyceride, 32.7% had high LDL, and 18.0% had low HDL. Waist circumference was significantly associated with having dyslipidemia. With every unit increase (1 cm), the odds of having dyslipidemia significantly increases by 1.020 (OR = 1.020, 95% CI (1.001, 1.039), *P*=0.041). Moreover, the odds of having dyslipidemia for patients with a waist circumference above the 90th percentile is significantly 2.760 times the odds of having dyslipidemia for patients below the 90th percentile (OR = 2.760, 95% CI (1.001, 7.615), *P*=0.05). As for standardized waist circumference (*z*-scores), every increase of 1 SD, the odds of having dyslipidemia significantly increases by 1.530 (OR = 1.530, 95% CI (1.120, 2.091), *P*=0.008) (Figure 1)

No significant associations with dyslipidemia were identified with age, gender, puberty stage, weight (absolute values), weight (*z*-scores), height (absolute value), height (*z*-scores), systolic, or diastolic blood pressure.

### 3.2. Elevated Liver Enzymes

Of the total 216 subjects, 210 had liver enzymes checked. 51 patients (24.3%) had either elevated AST or ALT or both (14.6% had high AST and 19.8% had high ALT).

A relationship between elevated liver enzymes with each of the following respective variables was found: age, weight (absolute value), and waist circumference. As age increases by 1 year, the odds of having elevated liver enzymes significantly increased by 1.142 (OR = 1.142, 95% CI (1.021, 1.278), *P*=0.020). Also, as weight (absolute value) increases by 1 kg, the odds of having elevated liver enzymes significantly increased by 1.013 (OR = 1.042, 95% CI (1.000, 1.025), *P*=0.042). Lastly, with every unit increase in waist circumference (1 cm), the odds of having elevated liver enzymes increase by 1.021. This finding is determined with a borderline significance (OR = 1.021, 95% CI (1.000, 1.043), *P* value = 0.052).

**Figure 2:**
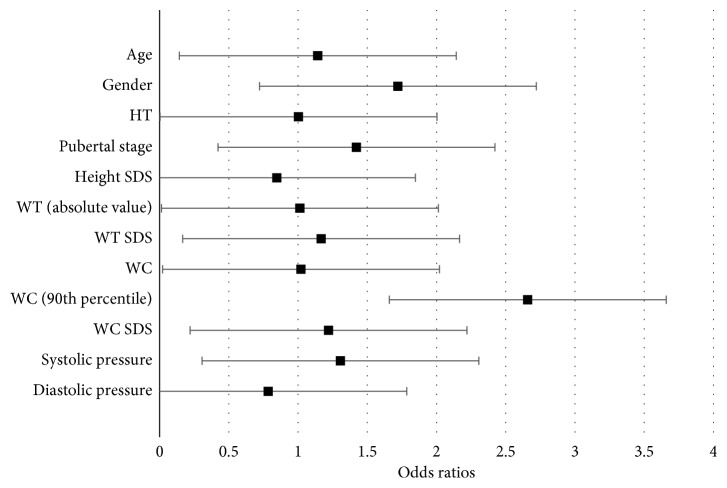
Bivariate logistic regressions between predictor variables and having elevated liver enzymes.

Gender, height (absolute value), puberty stage, height (*z*-scores), weight (*z*-scores), waist circumference (>90th percentile), waist circumference (*z*-scores), systolic pressure, and diastolic pressure were not significantly associated with elevated liver enzymes (Figure 2).

### 3.3. Fatty Liver Disease

**Figure 3:**
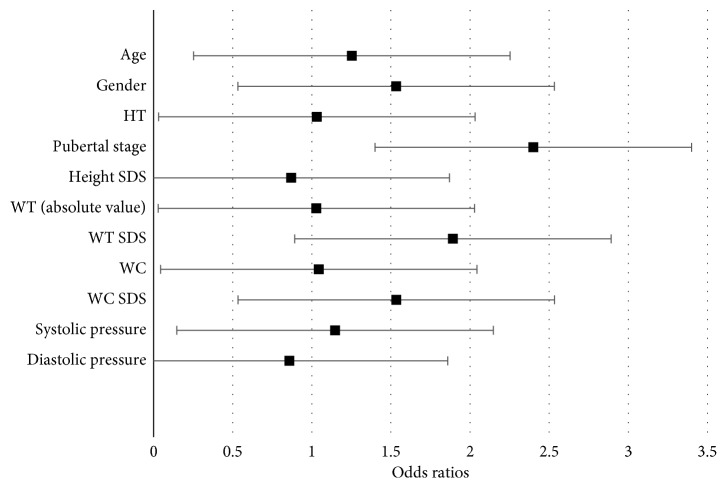
Bivariate logistic regressions between predictor variables and having fatty liver disease.

51 patients had high ALT or AST or both. All 51 patients had liver US. Of those, 43 had fatty liver (84%). Liver transaminases were lower in the 8 patients who did not have fatty liver. NAFLD was significantly associated with age, height (absolute value), weight (absolute value), weight (*z*-scores), waist circumference (absolute values), and waist circumference (*z*-scores) (Figure 3). As age increases by 1 year, the odds of having fatty liver significantly increase by 1.253 (OR = 1.253, 95% CI (1.072, 1.464), *P*=0.005). As height (absolute value) increases by 1 cm, the odds of having fatty liver significantly increase by 1.032 (OR = 1.032, 95% CI (1.003, 1.062), *P*=0.030). Moreover, as weight (absolute value) increases by 1 kg, the odds of having fatty liver significantly increase by 1.029 (OR = 1.029, 95% CI (1.013, 1.045), *P* < 0.001). Similarly, weight (*z*-scores) is also significantly associated with fatty liver (OR = 1.892, 95% CI (1.018, 3.518), *P* value = 0.044). As for waist circumference, both absolute value and *z*-scores are significantly associated with having fatty liver (OR = 1.044, 95% CI (1.015, 1.073), *P*=0.003; OR = 1.534, 95% CI (1.023, 2.301), *P*=0.038). Puberty staging showed a significant association with having a fatty liver with an OR of 3.35 (*P*=0.002) (Figure 3). The following variables were not significantly associated with having NAFLD: gender, height (*z*-scores), and systolic and diastolic blood pressure.

## 4. Discussion

Multiple studies have shown that obesity is one of the most important risk factors of metabolic disorders in children [15]. It was shown that 70% of obese or overweight children have at least one clinical cardiovascular risk factor, 52% of them have at least two risk factors, and 12% have three or more risk factors [3].

Researchers reported that 45.8% of the overweight children showed abnormal lipid profile. 10% of the children had high LDL levels, while 15% had increased LDL and TG with a higher prevalence in boys [17]. The results from our study revealed that, 32.7% of patients had high LDL, 18.0% had low HDL, and 28.6% of patients had high TG. Non-HDL cholesterol is a better indicator for persistent dyslipidemia and atherosclerosis in children and adults [18]. However, we were not able to run this analyte in our local lab.

In obese children, BMI has better practical utility in identifying dyslipidemia among school-aged children with obesity compared with other anthropometric measures [19]. While BMI is a useful marker for overall obesity, WC, waist-to-height ratio, and waist-to-hip ratio are more useful indices for defining abdominal obesity with WC showing the strongest relationship [20]. The International Diabetes Federation defined WC as one of the risk criteria for metabolic disorders in children [21]. In Guangzhou, China, BMI correctly identified 77% of the total dyslipidemic disorders in obese children, which was higher than that by waist hip ratio (WHR) (70.8%) (*P* < 0.05) [19]. In our study, results showed that dyslipidemia is significantly related to WC. This was in agreement with a study by Janssen et al. who reported that youths with high WC had high TG [22] and with another group of researchers who showed that the absolute WC had the strongest correlation with low HDL [13].

The probability of NAFLD increases with high WC. Studies showed that NAFLD is associated with larger WC [23]. Unlike gender, pubertal status, and blood pressure, age and weight were directly proportional with dyslipidemia and NAFLD.

Elevated liver enzyme (ALT) has been considered as an indicator for NAFLD in obese children and its elevation is considered as an indication for biopsy [10]. However, in recent practice guidelines, it was highlighted that significant histological abnormalities of NAFLD, including advanced fibrosis can be seen in children with normal or mildly elevated ALT levels. Accordingly, use of ALT alone may underestimate the extent of liver injury [24].

It is stated that liver US is a predictor of NAFLD [25]. In our sample, 84% of those who had elevated liver enzymes had features of NAFLD on liver US. Patients who had normal liver US had less elevation of liver enzymes compared to those who were shown to have NAFLD on scans. While liver enzymes are used to predict presence of NAFLD, their level was not shown to correlate with the histological grade of steatosis [9].

Findings from our study have also revealed various associations of demographic and antropometric measures. Dyslipidemia is shown to be significantly associated with WC. Elevated liver enzymes appear to be seen with increased age and waist circumference. We have seen an increased liver enzyme correlating with absolute weight but not weight SDS which might reflect an association with body mass rather than age-adjusted weight.

NAFLD is more prevalent in subjects with a higher WC and is directly proportional to age, weight, and height. We did not find any gender difference in relation to the frequency of the comorbidity. Puberty status did not appear to have an impact on predisposition to comorbidities either.

History of patient's family serves as an important predictor for the various comorbidities. Parental obesity could be considered as a key factor in the development of obesity due to genetic factors and family food choices [26]. In our sample, 80% of children had family history of obesity, 79% diabetes, and 55.5% had history of hyperlipidemia. This indicates the importance of genetics and geographical variations when studying the epidemiology of obesity and their comorbidities.

Study limitation is that it represents a single center in the UAE. Involving multiple centers throughout the country will give a more comprehensive data on these obesity comorbidities.

## 5. Conclusion

Dyslipidemia is seen frequently in overweight and obese children and is significantly related to WC. Similarly, there is a strong association between WC and NAFLD. This study provides an insight on the higher frequency of dyslipidemia and NAFLD in children and adolescent in our region where obesity is prevalent. The findings that emerged can be utilized to help further understanding of epidemiological gap related to obesity in children in the region. WC is an easy tool that can be utilized to screen for dyslipidemia in overweight and obese children and adolescents in community setting. Efforts need to be intensified towards addressing prevention strategies and raising awareness on obesity complications among youths.

## Figures and Tables

**Table 1:** Sample characteristics.

	Frequency (%)	Mean (SD)
*Demographics*		
Gender (male)	121 (56.0)	
Age		10.58 (2.996)

*Physical exam*		
Height-for-age		1.1950 ± 0.44
Weight-for-age		2.30 ± 0.704
WC-for-age		2.31 ± 1.058
WC ≥ 90th percentile	173 (89.6)	
Overweight	16 (7.0)	
Obese	200 (93.0)	
SBP-for-age		1.10 ± 1.014
DBP-for-age		0.55 ± 0.812
Acanthosis nigricans	138 (63.9)	

*Family history*		
Obesity	172 (80.0)	
Hyperlipidemia	117 (55.5)	
Heart disease	88 (40.7)	
Diabetes	170 (79.4)	

## Data Availability

The data used to support the findings of this study are available from the corresponding author upon request.
